# Tunable Optical Nanocavity of Iron-garnet with a Buried Metal Layer

**DOI:** 10.3390/ma8063012

**Published:** 2015-05-28

**Authors:** Alexey N. Kuz’michev, Lars E. Kreilkamp, Mohammad Nur-E-Alam, Evgeni Bezus, Mikhail Vasiliev, Iliya A. Akimov, Kamal Alameh, Manfred Bayer, Vladimir I. Belotelov

**Affiliations:** 1 Lomonosov Moscow State University, Moscow 119991, Russia; E-Mail: v.i.belotelov@ya.ru; 2 Russian Quantum Center, Skolkovo, Moscow Region 143025, Russia; 3 Experimentelle Physik 2, Technische Universitat Dortmund, D-44221 Dortmund, Germany; E-Mails: lars.kreilkamp@tu-dortmund.de (L.E.K.); ilja.akimov@tu-dortmund.de (I.A.A.); mmanbaye@googlemail.com (M.B.); 4 Electron Science Research Institute, Edith Cowan University, Joondalup, WA 6027, Australia; E-Mails: m.nur-e-alam@ecu.edu.au (M.N.-E-A.); m.vasiliev@ecu.edu.au (M.V.); k.alameh@ecu.edu.au (K.A.); 5 Image Processing Systems Institute, Russian Academy of Sciences, 443001 Samara, Russia; E-Mail: evgeni.bezus@gmail.com; 6 Samara State Aerospace University, 443086 Samara, Russia; 7 Ioffe Physical-Technical Institute, Russian Academy of Sciences, 194021 Saint Petersburg, Russia

**Keywords:** planar waveguides, surface plasmon-polariton, Faraday effect, gyrotropic media, optical nanocavity, metal nanocavity

## Abstract

We report on the fabrication and characterization of a novel magnetophotonic structure designed as iron garnet based magneto-optical nanoresonator cavity constrained by two noble metal mirrors. Since the iron garnet layer requires annealing at high temperatures, the fabrication process can be rather challenging. Special approaches for the protection of metal layers against oxidation and morphological changes along with a special plasma-assisted polishing of the iron garnet layer surface were used to achieve a 10-fold enhancement of the Faraday rotation angle (up to 10.8°/*µ*m) within a special resonance peak of 12 nm (FWHM) linewidth at a wavelength of 772 nm, in the case of a resonator with two silver mirrors. These structures are promising for tunable nanophotonics applications, in particular, they can be used as magneto-optical (MO) metal-insulator-metal waveguides and modulators.

## 1. Introduction

Optical nanocavities are powerful tools for controlling the interaction of light with matter and have been employed in a wide range of areas from quantum optical devices [1] to ultrasensitive optical sensors [2]. These applications rely on the effective confinement of light in a small cavity mode volume. In the area of nanophotonics, metal-dielectric [3,4], and dielectric cavity (or photonic crystal-based ) [5] waveguides are in widespread use today. On the other hand, the development of tunable structures which can provide ways to modify the optical properties in response to external stimuli is of prime importance nowadays. For this purpose, quantum dots [6], thermo-optic [7], acousto-optic [8], or electro-optic materials [9] have been utilized. Magneto-optical effects, such as Faraday rotation of light, are strongly enhanced in microcavities [10,11].

Incorporation of a ferromagnetic material into an optical cavity is very promising for achieving efficient control of light via an external magnetic field. So far, almost all magneto-optical resonators have been based on using a thin magnetic film sandwiched in-between two dielectric Bragg mirrors, and these structures are commonly referred to as 1D magnetophotonic crystals [12,13,14,15]. Magneto-optical cavities inside 2D and 3D periodic dielectric structures were studied as well [16,17]. Enhancement of the Faraday effect by an order of magnitude has been gained.

Magneto-optical cavities with metal walls have been considered only in a few papers [18,19,20,21]. Since at frequencies lower than the plasma frequency, metals have negative dielectric permittivity, using metallic cavity walls expands the available diversity of the cavity optical modes. In particular, surface plasmon polaritons can be excited in such structures [22]. Surface plasmon-polaritons constrained inside a metal cavity have been reported to be of interest for nanophotonics applications [23,24]. The authors of [19] experimentally studied stacks composed of a noble metal, ferromagnetic metal and nonmagnetic dielectric. An optimization of the layer thicknesses allowed for minimisation of the overall optical losses of the structure. However, it was only possible up to a certain point, since ferromagnetic metals are very lossy at optical frequencies. Minimization of optical absorption losses becomes crucial when the waveguiding properties of the magneto-optical cavities are of importance. In this respect, magnetic dielectrics like bismuth iron garnets are promising, since their absorption coefficient can be as low as 100 cm^−^^1^ or even smaller and their specific Faraday rotation is still rather large (near 1 °/*µm*) [25].

Significant enhancement in the MO effects due to the excitation of surface plasmon-polaritons (SPPs) and waveguide modes has been achieved in structures containing magnetic dielectrics [26,27,28,29,30,31,32]. The excitation of SPPs has been shown to provide a transversal magneto-optical Kerr effect as large as 15% in magnitude [26,29] and the excitation of hybrid plasmonic-waveguide modes gives rise to the novel magneto-optical intensity effect [33] that enables light intensity modulation at the level of 25% [34]. However, magneto-optical nanocavities with metal walls and magnetic dielectric layers have been investigated only theoretically [20,21]. The experimental demonstration of such structures has been impeded by several technological issues identified below.

In this work, we report on the design and fabrication of a novel multilayer structure for tunable nanophotonics which is promising for use in MO waveguides, as well as for the modulation of propagating optical waves. A unique feature of the reported structure is related to the presence of a buried metal layer placed under the magnetic dielectric that, together with an upper metal layer, constitutes a special type of magneto-optical nanocavity with metal walls. We managed to overcome the technological problems related to multi-material cavity formation and describe the method used for its fabrication, experimental measurements of transmittance and Faraday rotation and ellipticity spectra, and magnetic hysteresis behavior of this MO nanocavity, along with the theory calculation results. A remarkable enhancement of the Faraday effect magnitude within a spectral resonance in comparison with the non-resonant case is demonstrated.

## 2. Experimental

**Figure 1:**
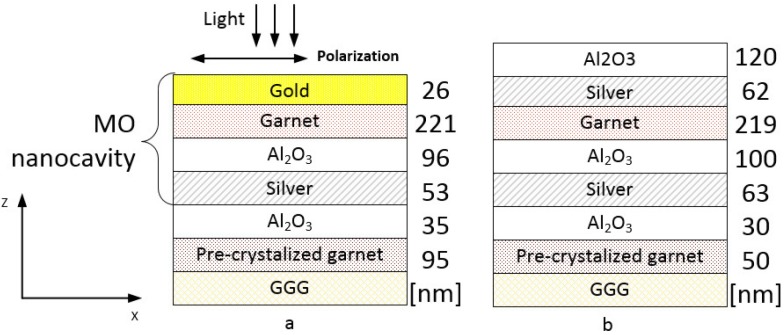
The schematics of the structure with: Buried Ag layer and Au layer on top (**a**), buried Ag layer and Ag, Al_2_O_3_ layers on top (**b**). For each sample thicknesses of the constitutive layers differ slightly. The numbers on the right side of each panel correspond to the layer thicknesses of (**a**) the sample-3 and (**b**) the sample-4.

The MO structure under consideration consists of an iron garnet film (Bi_2_Dy_1_Fe_4_Ga_1_O_12_) and Al_2_O_3_ layers surrounded by two nobel metal layers (gold or silver). The main technological obstacle for the implementation of this nanocavity type is related to the annealing of the iron garnet layer at temperatures higher than 650 °C that is required for making the iron garnet layer crystallize and thus acquire its ferrimagnetic nature. Moreover, running the annealing process near a specific temperature optimized for each garnet composition type is necessary for the garnet layer to have high specific Faraday rotation along with the uniaxial magnetic anisotropy and strong remanent properties. However, at the annealing process temperatures required to crystallize most garnets, the plasmonic metal layers (either gold or silver) lose their uniformity and morphological quality, and also tend to form into nanoparticles and therefore demonstrate surface degradation or oxidation (in case of Ag). We overcome this problem by introducing two Al_2_O_3_ dielectric bracket-type layers to help isolate the Ag layers chemically from the effects of diffusing oxygen and to keep them smooth, avoiding the oxidation-related composition changes Figure 1.

Apart from that, the entire multilayer structure is prepared on a partially-crystallised garnet layer. This layer is obtained by pre-annealing at lower temperatures and shorter times than is required for obtaining crystallised iron garnet. Its composition is selected to be the same as for the top garnet layer. This layer acts similarly to a “sacrificial oxide”. Namely, it captures the diffusing oxygen atoms migrating from the substrate pores towards the upper silver layer. If these diffusing oxygen atoms could complete the garnet’s stoichiometry (replacing some oxygen content loss that occurs typically during RF sputtering of oxide-mix-based garnet-stoichiometry targets) to make the garnet phase formation possible, then these atoms will not be able to travel further, to oxidise the metal(s). Using the partially-crystallised garnet-type layer instead of some other type of sacrificial oxide is advantageous; since the garnet phase formation is expected to occur actively at the same temperature as is actually being used during the annealing process of the main iron garnet layer. Therefore, there is a good likelihood of using up most of the diffusing oxygen in this additional garnet layer and prevent the oxidation of the buried silver.

At first, we fabricated two samples using this approach: Sample-1 and 2 Figure 1a. They differ only in the annealing process parameters utilized for their crystallization. For the sample-1, a multi-step annealing processes has been implemented, which was designed as a sequence of 30 min annealing at 600 °C followed by 90 min-long annealing at 630 °C, and finally, 650 °C was maintained for 90 min in the third stage of the annealing process. For the sample-2 a single-step annealing process has been used (650 °C for 4 h.) Influence of the annealing process on the garnet surface roughness and crystallinity has been studied by us previously [35]. In that paper we showed AFM and TEM images of the single- and multi-layer garnets before and after annealing. It follows from that studies that the annealing increases the surface roughness up to 20–40 nm. It is due to the appearance of crystalline grains of an average size of 50 nm. In order to compare the results with the non-resonant structure case, some parts of the sample-1 and -2 batches were kept, in which no upper layer of Au was deposited onto the garnet. We denote such samples as “not covered”: samples-1nc and 2nc. As it follows below, later sample-3 and sample-4 were obtained.

All magneto-optical measurements were performed at room temperature. In order to measure the optical spectra in a wide range of wavelengths, a white light source was used (Tungsten halogen lamp, Ocean Optics HL-2000-HP). Since a point source of light is necessary for homogeneous intensity distribution within the light spot, the lamp light was focused onto a pinhole of 100 *µ*m diameter. The incident light was focused in a light cone with an angle of below 1°. To achieve this, a tunable collimating pinhole was placed before the focusing lens with a focal length of 150 mm. The sample was placed in-between the poles of the electromagnet (AMT&C Group Ltd, London, UK) with a 40 mm gap. The external magnetic field was controlled by a computer and a power supply. The polarization of light was adjusted using a film-based linear polarizer (Thorlabs LPVIS050) providing an extinction ratio of 10^−^^5^. Light transmitted through the sample was collimated by an achromatic lens and focused onto the entrance slit of the spectrograph (Solar Laser Systems M266). The latter had a linear dispersion of 5.88 nm/mm. A Hamamatsu S10420-1106 2048 × 64-pixels back-thinned CCD image sensor with a spectral resolution of 0.3 nm was used.

## 3. Results and Discussion

**Figure 2:**
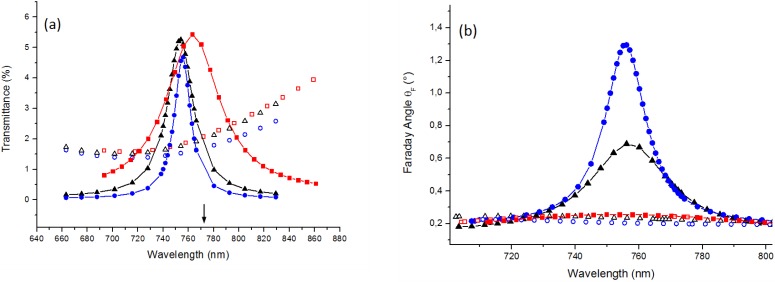
Transmittance spectra (**a**) and Faraday rotation spectra (**b**) for the nanocavity samples-1, 2 and 3 with Au layer on top (filled rectangles, triangles and circles, respectively) and for the “not covered” samples: Sample- 1nc, 2nc and 3nc (open rectangles, triangles, and circles, respectively). Arrow below indicates *λ_FP_* calculated from (1) for the sample-3.

The samples 1 and 2 exhibit spectrally-resonant features in their transmittance (Figure 2a) and Faraday rotation (Figure 2b) spectra. This is due to the excitation of the Fabry-Perot-type resonances. The observed resonance wavelength is in good agreement with the resonance wavelength given by the Fabry-Perot condition. The calculated resonance wavelength for the sample-3 is shown by an arrow in Figure 2a. The mismatch between the calculated and measured resonance wavelengths occurs due to the auxiliary layers present in the sample and layer interface roughness as will be explained further below):

(1) $$\lambda_{FP}=\frac{2(n_{1}h_{1}+n_{2}h_{2})}{\left(m-\frac{\delta \text{Φ}}{2\pi }\right)},$$

where *n*_1,2_ are refractive indices, and *h*_1,2_ are layer thicknesses of the iron garnet and Al_2_O_3_ layers, respectively, *δ*Φ is a phase shift occurring on reflection from the two metal layers, and *m* is an integer.

In terms of the resonance quality factor and Faraday effect enhancement, the sample-2 performance is much better in comparison with the sample-1. At the same time, the sample-1 gives only a marginal advantage over the non-resonant structure case. This can be explained by a much better layer-interface surface quality of the sample-2 which most likely resulted from a more suitable set of annealing conditions. The appearance of large roughness features on the garnet layer surfaces after running the annealing processes without following carefully-optimized thermal treatment regimes is (in our experience) a frequent occurrence, happening due to the formation of various non-garnet precipitate phases of materials on garnet-type layer surfaces.

**Figure 3:**
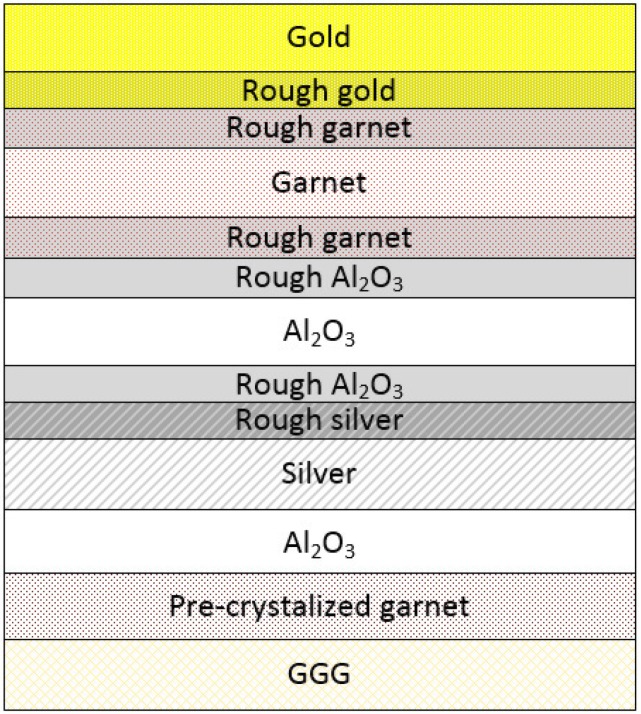
The schematics of the structure used for the modeling of the optical spectra of the samples-1, 2 and 3. The roughnesses of the gold, silver and iron garnet layers were taken into account by introducing “sublayers” (marked as “rough” gold, iron garnet, Al_2_O_3_ and silver, respectively) having the imaginary parts of their permittivity function increased *q_i_* times.

The presence of the surface roughness leads to increased Rayleigh scattering. Since scattering diminishes transmittance it can be taken into account effectively by increasing the imaginary part of the dielectric permittivity in a thin near-to-surface sublayer of a rough material. This approach has been introduced and successfully applied in [36,37,38] where a good agreement between modeling and experimental results was achieved. In order to avoid here any unnecessary complications we use a similar approach to identify the effects of the surface roughness on the optical properties of the fabricated samples. For that reason we introduced additional thin sublayers into the vicinity of the interfaces of each layer forming the nanocavity. The roughnesses of all interfaces not positioned within the nanocavity have been neglected. Therefore, such sublayers were introduced at the bottom of the Au layer, at both interfaces of the iron garnet layer, and also the aluminium oxide, as well as at the top of the buried Ag layer (Figure 3). The sublayer thicknesses *t_i_* were selected to be of the roughness scale and are described computationally by their dielectric permittivity function having its imaginary part increased by *q_i_* times. Several conditions on *t_i_* and *q_i_* were imposed: (i) *t_i_* of the adjacent rough layers were set to be equal and *q_i_* were assumed to be fixed for a given material for all four samples; (ii) the following limits for *q_i_* were set: 1 *< q_i_* *<* 5. The calculations were performed using the rigorous coupled-wave analysis (RCWA) method [39].

**Figure 4:**
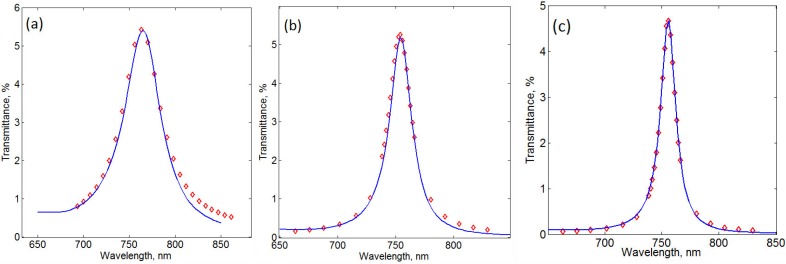
Transmittance spectra for the sample-1 with Au layer on top (**a**), the sample-2 with Au layer on top (**b**), and the sample-3 with Au layer on top (**c**). Solid lines show the calculation results, and the diamond shapes represent the experimental data. See in the text for the details on *t_i_* and *q_i_*.

For the sample-1, the best agreement between the calculation results and the experimental data was achieved using the following parameters: Au(smooth) 0 nm/Au(rough) 23 nm/ Garnet(rough) 23 nm/Garnet(smooth) 154 nm/Garnet(rough) 48 nm/Al_2_O_3_(rough) 48 nm/ Al_2_O_3_(smooth) 3 nm/Al_2_O_3_(rough) 45 nm/Ag(rough) 45 nm/Ag(smooth) 0 nm/Al_2_O_3_ 45 nm/ Garnet 55 nm, *q*(Al_2_O_3_) = 2.5, *q*(Ag) = 4.9, *q*(Garnet) = 3.2, *q*(Au) = 2.5 (Figure 4a). On the other hand, the optimal parameters for the modeling of the sample-2 were: Au(smooth) 17 nm/Au(rough) 7 nm/ Garnet(rough) 7 nm/Garnet(smooth) 203 nm/Garnet(rough) 10 nm/Al_2_O_3_(rough) 10 nm/ Al_2_O_3_(smooth) 77 nm/ Al_2_O_3_(rough) 9 nm/Ag(rough) 9 nm/Ag(smooth) 41 nm/Al_2_O_3_ 35 nm/ Garnet 95 nm, *q*(Al_2_O_3_) = 2.5, *q*(Ag) = 4.9, *q*(Garnet) = 3.2, *q*(Au) = 2.5 (Figure 4). It turned out that for obtaining the best matching between the experimental and calculated data, larger values of the rough sublayer thicknesses (for the same *q_i_* coefficients) were needed for the sample-1, compared with the sample-2. This is in agreement with our assumption that in order to obtain spectrally-sharp optical resonances, the layer surface interface quality are of extreme importance (the sample-2 is smoother than the sample-1). Since the rough-sublayer thicknesses are still rather large, the interface smoothness for both the metal and iron garnet layers still needs to be improved.

In order to make further progress, we polished the surfaces of the iron garnet layers using an argon plasma assisted ion bombardment process. For this, an oxide-base-mixed sputtering target (BaTiO_3_) in RF magnetron sputtering system with no extra oxygen or nitrogen input was used. The special ion-polishing procedure developed by our ECU-based group of co-authors allows smoothing of the layer surfaces by ablating the BaTiO_3_ target whilst avoiding the deposition of this material. The atomic force microscopy analysis proved decrease of the roughness through the plasma polishing by several times.

Sample-3 was obtained by using this plasma polishing procedure. As follows from Figure 2a,b, this sample has demonstrated a much better spectral performance. In particular, it provided 1.25° of Faraday rotation at 756 nm. This corresponds to an almost 6-fold enhancement in comparison with the non-resonant structure case (compared with the sample-3nc). The full width at half maximum (FWHM) for this resonance peak is only 18 nm. It appears that the constituting layers of the sample-3 are much smoother than in the previous two samples. This conclusion is also supported by the calculations that result in the best matching with the experimental data for the following sublayer parameter sets: Au(smooth) 21 nm/Au(rough) 5 nm/Garnet(rough) 5 nm/Garnet(smooth) 209 nm/Garnet(rough) 7 nm/ Al_2_O_3_(rough) 7 nm/Al_2_O_3_(smooth) 83 nm/Al_2_O_3_(rough) 6 nm/Ag(rough) 6 nm/Ag(smooth) 47 nm/ Al_2_O_3_ 35 nm/Garnet 95 nm, *q*(Al_2_O_3_) = 2.5, *q*(Ag) = 4.9, *q*(Garnet) = 3.2, *q*(Au) = 2.5 (Figure 4c).

**Figure 5:**
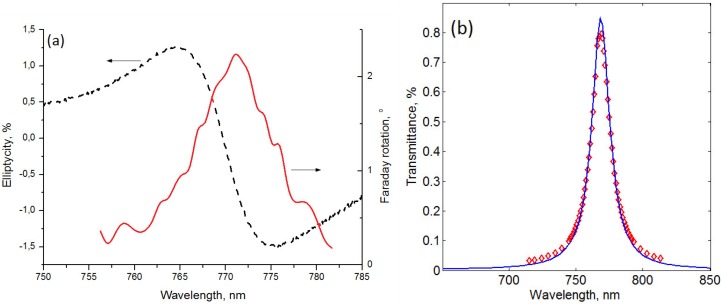
(**a**) Experimental Faraday rotation (solid line) and Faraday ellipticity spectra (dashed line) for the sample-4 with Ag layer on top. (**b**) Transmittance spectra for the sample-4 with Ag layer on top. Solid line is for the calculation and diamonds represent experimental data.

**Figure 6:**
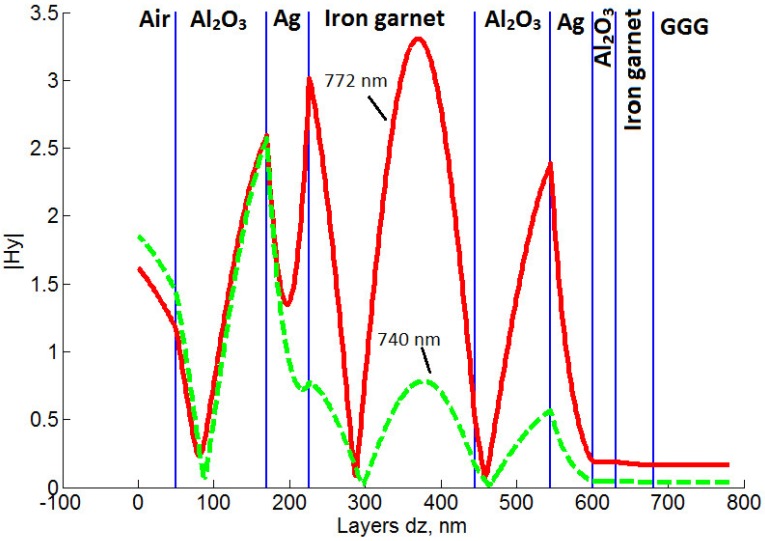
|H*_y_*| component of light field intensity distribution for the sample-4: At the resonance wavelength of 772 nm (solid red line) and at the non-resonant wavelength of 740 nm (dash green line).

Using the best-known annealing process parameters along with the new argon plasma assisted ion bombardment process, another sample (sample-4) with two silver layers was produced, having even better optical performance (Figure 1b). This sample differs from the previous ones by the substitution of the top gold layer with a silver and the addition of Al_2_O_3_ layer on top. The dielectric oxide was placed on top of the Ag layer in order to prevent silver from entering into any chemical reactions and to prevent mechanical damage. This sample demonstrates even better magneto-optical properties than the sample-3. It provides 2.3° of Faraday rotation at 772 nm (Figure 5a) which is 1.8 times larger in comparison with the sample-3. The FWHM linewidth for the resonance peak of the sample-4 is only 12 nm, whereas the sample-3 has a 1.5 times wider FWHM linewidth. However, the transmittance spectrum measurements (Figure 5b) show less percentage of the transmitted light intensity than in the previous samples, due to thicker metal layers used: Al_2_O_3_ 120 nm/Ag(smooth) 55 nm/ Ag(rough) 7 nm/Garnet(rough) 7 nm/Garnet(smooth) 204 nm/Garnet(rough) 8 nm/Al_2_O_3_(rough) 8 nm/ Al_2_O_3_(smooth) 86 nm/Al_2_O_3_(rough) 6 nm/Ag(rough) 6 nm/Ag(smooth) 57 nm/Al_2_O_3_ 30 nm/ Garnet 50 nm, *q*(Al_2_O_3_) = 2.5, *q*(Ag) = 4.9, *q*(Garnet) = 3.2. As discussed above, the enhancement of the Faraday effect is due to the Fabry-Perot resonance. This is also supported by the electromagnetic field distribution (amplitude of *H_y_* field component) inside layers of the sample-4 at the resonance wavelength *λ* = 772 nm and at a wavelength *λ* = 740 nm away from the resonance (Figure 6). Significant concentration of the electromagnetic energy is observed within the main magnetic layer of the sample-4 in the resonant case.

For the sample-4, the transmitted light ellipticity was measured as well (Figure 5a). The ellipticity is defined as the ratio between the minor and major axes of the polarization ellipse. As usual, it vanishes at the peak of the Faraday rotation and has maxima at its slopes.

**Figure 7:**
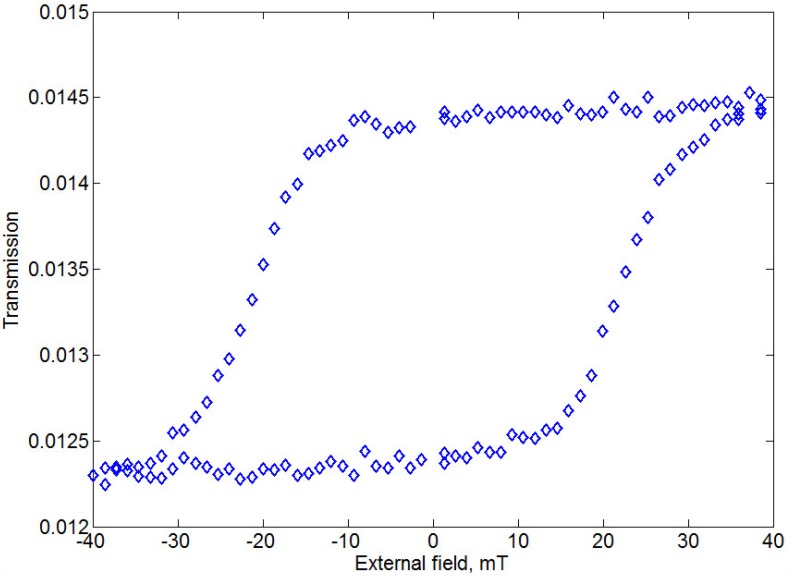
Transmittance-mode Faraday hysteresis loop for the sample-4 with Ag layer on top.

The magnetic hysteresis was measured in Faraday configuration with crossed polarizers (at *λ* = 772 *nm*) (Figure 7). The saturation of the iron garnet magnetization takes place at relatively low magnetic field of 40 mT. Also, magnetic remanence properties are quite well pronounced: Such structure provides in zero magnetic field almost the same Faraday rotation as for the saturation regime.

## 4. Conclusions

To conclude, we demonstrated a novel type of magneto-optical nanocavities with magnetic dielectric (bismuth iron garnet) central layer and two metal (Au or Ag) mirrors. Technological obstacles affecting the structure fabrication process were successfully overcome. Optical and magneto-optical characterization revealed that a special approach for the chemical isolation of the buried Ag layer along with the plasma-assisted polishing of the iron garnet layer surface led to achieving a specific Faraday rotation angle of 5.8°/*µ*m within a spectral resonance peak of 18 nm FWHM linewidth. Even better performance was obtained in the sample constructed as a magneto-optical nanocavity with twin Ag mirrors—A Faraday rotation of 10.8°/*µ*m within a narrow peak of 12 nm FWHM linewidth was demonstrated at 772 nm. The magneto-optical nanocavity structures might find applications in tunable nanophotonics including gyrotropic waveguides and magneto-optical modulators.

## References

[1] Vahala K.J. (2003). Optical microcavities. Nature.

[2] Armani A.M., Kulkarni R.P., Fraser S.E., Flagan R.C., Vahala K.J. (2007). Label-free, Single-molecule detection with optical microcavities. Science.

[3] Dionne J.A., Sweatlock L.A., Atwater H.A., Polman A. (2006). Plasmon slot waveguides: Towards chip-scale propagation with subwavelength-scale localization. Phys. Rev. B.

[4] Zia R., Selker M.D., Catrysse P.B., Brongersma M.L. (2004). Geometries and materials for subwavelength surface plasmon modes. J. Opt. Soc. Am. A.

[5] Vasiliev M., Kotov V.A., Alameh K.E., Belotelov V.I., Zvezdin A.K. (2008). Novel magnetic photonic crystal structures for magnetic field sensors and visualizers. IEEE Trans. Magn..

[6] Pacifici D., Lezec H.J., Atwater H.A. (2007). All-optical modulation by plasmonic excitation of CdSe quantum dots. Nat. Photonics.

[7] Nikolajsen T., Leosson K., Bozhevolnyi S.I. (2004). Surface plasmon polariton based modulators and switches operating at telecom wavelengths. Appl. Phys. Lett..

[8] Jory M.J., Bradberry G.W., Cann P.S., Sambles J.R. (1995). A surface-plasmon-based optical sensor using acousto-optics. Meas. Sci. Technol..

[9] Dionne J.A., Diest K., Sweatlock L.A., Atwater H.A. (2009). PlasMOStor: A Metal-Oxide-Si field effect plasmonic modulator. Nano Lett..

[10] Kaliteevskii M.A., Kavokin A.V., Kop’ev P.S. (1997). Faraday rotation of light in a microcavity. Semiconductors.

[11] Kavokin A.V., Vladimirova M.R., Kaliteevski M.A., Lyngnes O., Berger J.D., Gibbs H.M., Khitrova G. (1997). Resonant Faraday rotation in a semiconductor microcavity. Phys. Rev. B.

[12] Koba M., Suffczynski J. (2013). Magneto-optical effects enhancement in DMS layers utilizing 1-D photonic crystal. J. Electromagn. Waves Appl..

[13] Inui C., Ozaki S., Kura H., Sato T. (2011). Enhancement of Faraday effect in one-dimensional magneto-optical photonic crystal including a magnetic layer with wavelength dependent off-diagonal elements of dielectric constant tensor. J. Magn. Magn. Mater..

[14] Baryshev A.V., Merzlikin A.M., Inoue M. (2013). Efficiency of optical sensing by a plasmonic photonic-crystal slab. Phys. D Appl. Phys..

[15] Vasiliev M., Kotov V.A., Alameh K., Belotelov V.I., Zvezdin A.K. (2011). Novel magnetic photonic crystal structures for magnetic field sensors and visualizers. IEEE Trans. Magn..

[16] Dmitriev V., Kawakatsu M.N., Portela G. (2013). Compact optical switch based on 2D photonic crystal and magneto-optical cavity. Opt. Lett..

[17] Maka T., Chigrin D.N., Romanov S.G., Sotomayor Torres C.M. (2003). Three dimensional photonic crystals in the visible regime. Prog. Electromagn. Res..

[18] Ferreiro-Vila E., Garcia-Martin J.M., Cebollada A., Armelles G., Gonzalez M.U. (2013). Magnetic modulation of surface plasmon modes in magnetoplasmonic metal-insulator-metal cavities. Opt. Express.

[19] Banthi J.C., Meneses-Rodriguez D., Garcia F., Gonzalez M.U., Garcia-Martin A., Cebollada A., Armelles G. (2012). High magneto-optical activity and low optical losses in metal-dielectric Au/Co/Au-SiO_2_ magnetoplasmonic nanodisks. Adv. Mater..

[20] Rukhlenko I.D., Premaratne M., Agrawal G.P. (2012). Guided plasmonic modes of anisotropic slot waveguides. Nanotechnology.

[21] Nikolova D., Fisher A.J. (2013). Switching and propagation of magnetoplasmon polaritons in magnetic slot waveguides and cavities. Phys. Rev. B.

[22] Raether H. (1986). Surface Plasmons on Smooth and Rough Surfaces and on Gratings.

[23] Belotelov V.I., Kalish A.N., Zvezdin A.K., Gopal A.V., Vengurlekar A.S. (2012). Fabry-Perot plasmonic structures for nanophotonics. J. Opt. Soc. Am. B.

[24] Abhilash T., Balasubrahmaniyam M., Patra A., Kasiviswanathan S. (2014). Plasmon resonance mediated enhancement in Fabry-Perot cavity modes. Appl. Phys. Lett..

[25] Zvezdin A.K., Kotov V.A., Coey J.M.D., Tilley D.R. (1997). Modern Magnetooptics and Magnetooptical Materials.

[26] Pohl M., Kreilkamp L.E., Belotelov V.I., Akimov I.A., Kalish A.N., Khokhlov N.E., Yallapragada V.J., Gopal A.V., Nur-E-Alam M., Vasiliev M. (2013). Tuning of the transverse magneto-optical Kerr effect in magneto-plasmonic crystals. New J.f Phys..

[27] Belotelov V.I., Bykov D.A., Doskolovich L.L., Kalish A.N., Kotov V.A., Zvezdin A.K. (2009). Giant magnetooptical orientational effect in plasmonic heterostructures. Opt. Lett..

[28] Belotelov V.I., Bykov D.A., Doskolovich L.L., Kalish A.N., Zvezdin A.K. (2010). Giant transversal Kerr effect in magnetoplasmonic heterostructures. J. Exp. Theor. Phys..

[29] Kreilkamp L.E., Belotelov V.I., Chin J.Y., Neutzner S., Dregely D., Wehlus T., Akimov I.A., Bayer M., Stritzker B., Giessen H. (2013). Waveguide-plasmon polaritons enhance transverse magneto-optical Kerr effect. Phys. Rev. X.

[30] Chekhov A.L., Krutyanskiy V.L., Shaimanov A.N., Stognij A.I., Murzina T.V. (2014). Wide tunability of magnetoplasmonic crystals due to excitation of multiple waveguide and plasmon modes. Opt. Express.

[31] Golovastikov N.V., Bykov D.A., Doskolovich L.L., Bezus E.A. (2015). Spatial optical integrator based on phase-shifted Bragg gratings. Opt. Commun..

[32] Belotelov V.I., Doskolovich L.L., Kotov V.A., Bezus E.A., Bykov D.A., Zvezdin A.K. (2007). Magnetooptical effects in the metal-dielectric gratings. Opt. Commun..

[33] Belotelov V.I., Kreilkamp L.E., Kalish A.N., Akimov I.A., Bykov D.A., Kasture S., Yallapragada V.J., Gopal A.V., Grishin A.M., Khartsev S.I. (2014). Magnetophotonic intensity effects in hybrid metal-dielectric structures. Phys. Rev. B.

[34] Belotelov V.I., Kreilkamp L.E., Akimov I.A., Kalish A.N., Bykov D.A., Kasture S., Yallapragada V.J., Gopal A.V., Grishin A.M., Khartsev S.I. (2013). Plasmon-mediated magneto-optical transparency. Nat. Commun..

[35] Vasiliev M., Wo P.C., Alameh K., Munroe P., Xie Z., Kotov V.A., Burkov V.I. (2009). Microstructural characterization of sputtered garnet materials and all-garnet magnetic heterostructures: Establishing the technology for magnetic photonic crystal fabrication. J. Phys. D.

[36] Dyakov S.A., Baldycheva A., Perova T.S., Li G.V., Astrova E.V., Gippius N.A., Tikhodeev S.G. (2012). Surface states in the optical spectra of two-dimensional photonic crystals with various surface terminations. Phys. Rev. B.

[37] Benisty H., Labilloy D., Weisbuch C., Smith C.J.M., Krauss T.F., Cassagne D., B‘eraud A., Jouanin C. (2000). Radiation losses of waveguide-based two-dimensional photonic crystals: Positive role of the substrate. Appl. Phys. Lett..

[38] Benisty H., Lalanne P., Olivier S., Rattier M., Weisbuch C., Smith C.J.M., Krauss T.F., Jouanin C., Cassagne D. (2002). Finite-depth and intrinsic losses in vertically etched two-dimensional photonic crystals. Opt. Quantum Electron..

[39] Gaylord T.K., Pommet D.A., Grann E.B. (1995). Stable implementation of the rigorous coupled-wave analysis for surface-relief gratings: enhanced transmittance matrix approach. J. Opt. Soc. Am. A.

